# Impact of mepolizumab on the AAV-PRO questionnaire in eosinophilic granulomatosis with polyangiitis: data from a European multicentre study

**DOI:** 10.1093/rheumatology/keaf232

**Published:** 2025-04-26

**Authors:** Paolo Delvino, Luca Quartuccio, Joanna C Robson, Virginia V Ferretti, Catherine Klersy, Federico Alberici, Diego Bagnasco, Alvise Berti, Marco Caminati, Marta Camoni, Maria C Cid, Edoardo Conticini, Giulia Costanzo, Claire de Moreuil, Stefano Del Giacco, Georgina Espigol-Frigole, Franco Franceschini, Luca Iorio, Anna Kernder, Alberto Lo Gullo, Laura Losappio, Elena Manna, Matteo Maule, Alessandra Milanesi, Carlomaurizio Montecucco, Simone Negrini, Roberto Padoan, Francesca Regola, Luisa Ricciardi, Jan Schroeder, Benjamin Terrier, Paola Toniati, Francesca Torracca, Elena Treppo, Maria Letizia Urban, Augusto Vaglio, Giacomo Emmi, Sara Monti, European EGPA Study Group, Paolo Delvino, Luca Quartuccio, Joanna C Robson, Virginia V Ferretti, Catherine Klersy, Federico Alberici, Diego Bagnasco, Alvise Berti, Marco Caminati, Marta Camoni, Maria C Cid, Edoardo Conticini, Giulia Costanzo, Claire de Moreuil, Stefano Del Giacco, Georgina Espigol-Frigole, Franco Franceschini, Luca Iorio, Anna Kernder, Alberto Lo Gullo, Laura Losappio, Elena Manna, Matteo Maule, Alessandra Milanesi, Carlomaurizio Montecucco, Simone Negrini, Roberto Padoan, Francesca Regola, Luisa Ricciardi, Jan Schroeder, Benjamin Terrier, Paola Toniati, Francesca Torracca, Elena Treppo, Maria Letizia Urban, Augusto Vaglio, Giacomo Emmi, Sara Monti

**Affiliations:** School of Medicine and Surgery, University of Milano-Bicocca, Milan, Italy; Rheumatology Unit, Fondazione IRCSS San Gerardo dei Tintori, Monza, Italy; Division of Rheumatology, Department of Medicine (DMED), Academic Hospital “Santa Maria della Misericordia”, ASUFC, University of Udine, Udine, Italy; School of Health and Social Wellbeing, University of the West of England, Bristol, UK; Rheumatology Department, University Hospitals Bristol and Weston NHS Foundation Trust, Bristol, UK; Biostatistcs and Clinical Trial Center, Fondazione IRCCS Policlinico San Matteo, Pavia, Italy; Biostatistcs and Clinical Trial Center, Fondazione IRCCS Policlinico San Matteo, Pavia, Italy; Department of Medical and Surgical Specialties, Radiological Sciences and Public Health, University of Brescia, Brescia, Italy; Nephrology Unit, Spedali Civili Hospital, ASST Spedali Civili of Brescia, Brescia, Italy; Allergy and Respiratory Diseases, Department of Internal Medicine (DIMI), IRCCS Policlinico San Martino, University of Genoa, Genoa, Italy; Center for Medical Sciences (CISMed), Department of Cellular, Computational and Integrative Biology (CIBIO), University of Trento, Trento, Italy; Unit of Rheumatology, S. Chiara Hospital, APSS, Trento, Italy; Department of Medicine, University of Verona, Verona, Italy; Asthma Center and Allergy Unit, Verona Integrated University Hospital, Verona, Italy; Department of Medical and Surgical Specialties, Radiological Sciences and Public Health, University of Brescia, Brescia, Italy; Nephrology Unit, Spedali Civili Hospital, ASST Spedali Civili of Brescia, Brescia, Italy; Department of Autoimmune Diseases, Hospital Clínic de Barcelona, University of Barcelona, Institut d'Investigacions Biomèdiques August Pi i Sunyer (IDIBAPS), Barcelona, Spain; Rheumatology Unit, Department of Medicine, Surgery and Neurosciences, University of Siena, Siena, Italy; Department of Medical Sciences and Public Health, University of Cagliari, Cagliari, Italy; Department of Internal Medicine, CHU Brest, UMR 1304 (GETBO), Brest, France; Department of Medical Sciences and Public Health, University of Cagliari, Cagliari, Italy; Department of Autoimmune Diseases, Hospital Clínic de Barcelona, University of Barcelona, Institut d'Investigacions Biomèdiques August Pi i Sunyer (IDIBAPS), Barcelona, Spain; Rheumatology and Clinical Immunology Unit, Dipartimento Continuità di Cure e Fragilità, ASST Spedali Civili di Brescia, Brescia, Italy; Department of Clinical and Experimental Sciences, University of Brescia, Brescia, Italy; Division of Rheumatology, Department of Medicine DIMED, University of Padua, Padova, Italy; Clinic for Rheumatology and Hiller Research Center, University Hospital Düsseldorf, Medical Faculty of Heinrich-Heine-University, Düsseldorf, Germany; Rheumazentrum Ruhrgebiet, Ruhr University Bochum, Herne, Germany; Rheumatology Unit, Arnas Garibaldi Hospital, Catania, Italy; Division of Allergy and Clinical Immunology, ASST GOM Niguarda, Milan, Italy; Department of Internal Medicine and Therapeutics, Università di Pavia, Pavia, Italy; Division of Rheumatology, Fondazione IRCCS Policlinico San Matteo, Pavia, Italy; Department of Medicine, University of Verona, Verona, Italy; Asthma Center and Allergy Unit, Verona Integrated University Hospital, Verona, Italy; Department of Internal Medicine and Therapeutics, Università di Pavia, Pavia, Italy; Division of Rheumatology, Fondazione IRCCS Policlinico San Matteo, Pavia, Italy; PhD in Experimental Medicine, University of Pavia, Pavia, Italy; Department of Internal Medicine and Therapeutics, Università di Pavia, Pavia, Italy; Division of Rheumatology, Fondazione IRCCS Policlinico San Matteo, Pavia, Italy; Internal Medicine, Clinical Immunology and Translational Medicine Unit, IRCCS Ospedale Policlinico San Martino, Genoa, Italy; Department of Internal Medicine (DIMI), University of Genoa, Genoa, Italy; Division of Rheumatology, Department of Medicine DIMED, University of Padua, Padova, Italy; Rheumatology and Clinical Immunology Unit, Dipartimento Continuità di Cure e Fragilità, ASST Spedali Civili di Brescia, Brescia, Italy; Department of Clinical and Experimental Sciences, University of Brescia, Brescia, Italy; Allergy and Immunology Unit, Department of Clinical and Experimental Medicine, G. Martino Teaching Hospital, University of Messina, Messina, Italy; Division of Allergy and Clinical Immunology, ASST GOM Niguarda, Milan, Italy; Department of Internal Medicine, National Referral Center for Rare Systemic Autoimmune Diseases, Hôpital Cochin, Paris, France; Paris Cité University, Paris, France; Rheumatology and Clinical Immunology Unit, Dipartimento Continuità di Cure e Fragilità, ASST Spedali Civili di Brescia, Brescia, Italy; APACS APS ETS—Associazione Pazienti Sindrome di Churg Strauss EGPA, Arosio, Italy; Division of Rheumatology, Department of Medicine (DMED), Academic Hospital “Santa Maria della Misericordia”, ASUFC, University of Udine, Udine, Italy; Department of Experimental and Clinical Medicine, University of Florence, Florence, Italy; Department of Biomedical, Experimental and Clinical Sciences ‘Mario Serio’, University of Firenze, Firenze, Italy; Nephrology and Dialysis Unit, Meyer Children's Hospital IRCCS, Firenze, Italy; Department of Medical, Surgery and Health Sciences, University of Trieste, Trieste, Italy; Clinical Medicine and Rheumatology Unit, Cattinara University Hospital, Trieste, Italy; Immunorheumatology Research Laboratory, IRCCS Istituto Auxologico Italiano, Milan, Italy

**Keywords:** eosinophilic granulomatosis with polyangiitis, mepolizumab, health-related quality of life, patient-reported outcomes, ANCA-associated vasculitis patient-reported outcomes questionnaire

## Abstract

**Objectives:**

To prospectively evaluate the impact and the rapidity of the effect of mepolizumab on the ANCA-associated vasculitis patient-reported outcomes (AAV-PRO) questionnaire and patient global assessment (PtGA) in an international, multicentre cohort of patients with eosinophilic granulomatosis with polyangiitis (EGPA).

**Methods:**

Patients with active EGPA initiating treatment with mepolizumab were included. PtGA and the AAV-PRO score were assessed at baseline and after 7, 14, 30, 90 and 180 days. Predictors of response of the AAV-PRO questionnaire were investigated.

**Results:**

Seventy patients were included: female 54.3%, median age 56 years (48–65), 63 (90%) with a relapsing/refractory course. PtGA showed a statistically significant decrease within 7 days. At 14 days, all the AAV-PRO domains, except treatment side effects, showed a statistically significant decline. The improvement at 6 months was greatest in organ-specific symptoms (ratio 0.53), physical function (ratio 0.57) and PtGA (ratio 0.58). PtGA and higher disease activity positively correlated with the AAV-PRO scores throughout the study. Female patients reported a greater burden in terms of systemic symptoms, treatment side effects, social and emotional impact and concerns about the future. Conversely, age, educational level, damage accrual, mepolizumab dose and ANCA status had no effect on the AAV-PRO scores.

**Conclusions:**

Mepolizumab was associated with a quick and remarkable improvement of health-related quality of life in patients with EGPA. These findings highlight its early and sustained benefits beyond disease control and support the integration of the AAV-PRO questionnaire into routine clinical practice.

Rheumatology key messages

- Mepolizumab rapidly improves the AAV-PRO scores in EGPA, with significant benefits within 2 weeks.
- Organ-specific symptoms, physical function and patient global assessment show the strongest improvement at six months.
- The AAV-PRO questionnaire provides valuable insights into treatment response beyond traditional disease activity measures.

## Introduction

Eosinophilic granulomatosis with polyangiitis (EGPA) belongs to anti-neutrophil cytoplasmic antibody (ANCA)-associated vasculitis (AAV), a group of rare disorders characterized by necrotizing inflammation of small and medium-sized blood vessels. The clinical picture of EGPA is heterogeneous, including late-onset asthma, chronic rhinosinusitis with nasal polyps (CRSwNP), hypereosinophilia and organ disfunction due to vasculitis and/or eosinophilic tissue infiltration [1–5]. Therapeutic approach to EGPA traditionally relied on the use of long-term glucocorticoids (GCs) and immunosuppressive agents. However, disease relapses and GC-dependency are common and require the long-lasting use of these medications, contributing to a considerable rate of adverse events (AEs) [6, 7]. Recently, targeted biological therapies have emerged [8]. Mepolizumab, a monoclonal antibody binding interleukin-5, proved its efficacy in the randomized MIRRA trial and in observational studies [9–12], providing a significant GC-sparing effect and a better control of respiratory symptoms. Based on this evidence, mepolizumab is currently recommended for the treatment of non-severe EGPA [13, 14]. Despite improved survival in AAV, disease chronicity—driven by relapses, sequelae and treatment-related toxicity—still represents a major concern [15, 16]. In the past few years, there has been growing interest in embedding patient-reported outcomes (PROs) in the assessment of AAV [17]. Indeed, the evaluation of PROs is of utmost importance to capture the full impact of the disease and its treatment from the patient’s perspective, providing valuable insights into several aspects of their health-related quality of life (HRQoL), such as symptom burden, treatment AEs and functional status [18]. The AAV-PRO questionnaire is a novel, 29-item, disease-specific tool, designed to measure HRQoL in AAV patients. It was validated in a large cohort of patients from the UK and USA and subsequently endorsed by the Outcome Measures in Rheumatology (OMERACT) Vasculitis Working Group in 2017 for use in clinical trials [19, 20]. A recent systematic review evaluating the psychometric properties of 22 outcome measurement instruments in AAV identified the AAV-PRO as the most validated tool to appraise HRQoL [21]. However, the impact of specific treatments for EGPA on PRO measures, including the AAV-PRO questionnaire, is still unclear. The aim of this study was to investigate (i) the influence and the rapidity of the effect of mepolizumab on the AAV-PRO questionnaire and patient global assessment (PtGA) in a multicentre European cohort of patients with EGPA; and (ii) the predictors and correlates of response of each AAV-PRO domain after the start of mepolizumab.

## Patients and methods

### Study design and population

We conducted a multicentre, prospective, observational study including consecutive patients with EGPA from 17 European referral centres for systemic vasculitis belonging to the European EGPA Study Group across four European countries (Italy, France, Spain, Germany) and initiating mepolizumab between January 2021 and June 2022. The study was approved by the Ethics Committee of Pavia (Comitato Etico di Pavia *–* Protocol Number: 0099944/21), in accordance with national legislation. Each patient signed informed consent for clinical and laboratory data for study purposes.

EGPA was classified using the 2022 ACR/EULAR classification criteria [22, 23] and/or the MIRRA trial proposed criteria [9]. Mepolizumab was administered at a dose of 100 mg/4 weeks or 300 mg/4 weeks, in accordance with local practice; data were collected before the regulatory approval of high-dose mepolizumab for EGPA. Patients were eligible for inclusion if they maintained a stable GCs dose for ≥2 weeks before enrolment. Concomitant immunosuppressive therapy was allowed, provided the dose was unchanged in the preceding 3 months. Patients with <6 months of follow-up were excluded. The ‘Associazione Pazienti Sindrome di Churg-Strauss – EGPA’ [24] was involved in corroborating the relevance of the AAV-PRO questionnaire and PtGA and informing timing of data collection, thereby ensuring alignment with patient-prioritized HRQoL domains.

### Data collection

Information collected at mepolizumab initiation included demographics, clinical characteristics, damage accrual, laboratory findings and treatment-related data. Laboratory findings, including blood eosinophil count and C-reactive protein (CRP), were reassessed after 1, 3 and 6 months from mepolizumab initiation.

### Disease activity, definition of remission and relapses, damage accrual

Disease activity was assessed using the Birmingham Vasculitis Activity Score (BVAS, version 3) [25], with clinical response being evaluated according to the BVASv3 and GCs dose. Remission was defined as BVASv3 = 0 and prednisone-equivalent dose ≤4 mg/day. Disease remission off-GCs was defined as BVASv3 = 0 and prednisone-equivalent dose = 0. Disease relapse was identified by the reappearance/worsening of disease activity, with a BVASv3 >0 and the need to increase GCs and/or to intensify immunosuppressive treatment. Relapses were differentiated between vasculitic relapses and those limited to asthma/ear, nose and throat (ENT), and between major and minor based on the presence of organ/life-threatening manifestations. Disease activity and relapses were evaluated at baseline and after 1, 3 and 6 months of treatment. Damage accrual was measured at baseline and after 6 months using the Vasculitis Damage Index (VDI) [26].

### The AAV-PRO questionnaire and PtGA

HRQoL was assessed using the AAV-PRO questionnaire and PtGA at baseline and at 1 week, 2 weeks, 1 month, 3 months and 6 months following mepolizumab initiation.

The AAV-PRO questionnaire includes 29 items, distributed in six different outcome domains (Supplementary Table S1, available at *Rheumatology* online): (i) ‘organ-specific symptoms’ (five items); ‘systemic symptoms’ (four items); ‘treatment side effects’ (five items); ‘social and emotional impact’ (six items); ‘concerns about the future’ (five items); and ‘physical function’ (four items). Each item has five response options scored from 0 to 4, with higher score representing greater severity. The raw score of each domain was obtained by summing the scores for each of the individual items’ responses. The 0–100 scale score for each domain was then calculated by multiplying the actual raw score by 100, divided by the maximum possible raw score for the domain [19]. All patients completed the AAV-PRO questionnaire in their native language. The Italian, French, German and Spanish versions employed in this study were developed according to approved methodology for translation of PRO measures. Steps included forward and backwards translation, inventor check for fidelity of translation compared with the original version, and five cognitive interviews with patients with AAV to check the translation was understandable and relevant to them. This ensured integrity of the measure and faithfulness of the translation.

PtGA was assessed on 100 mm visual analogue scale (VAS) with the question ‘Please mark the line below indicating how active you believe your EGPA has been in the past 7 days’. Patients were instructed to express only the impact of the disease itself, and not that of other medical conditions or treatment AEs. Higher PtGA scores were intended as higher levels of perceived disease activity.

### Study endpoints and statistical analysis

#### Endpoints

The primary endpoint (endpoint 1) was to evaluate changes in the 0–100 score of the six AAV-PRO domains and PtGA over the 6-month observation period following mepolizumab initiation. Secondary endpoints included: the association of each of the AAV-PRO domains with PtGA, disease activity and damage accrual (endpoint 2.1); the influence of baseline demographics, clinical and biological factors on the performance of the 6 AAV-PRO domains over time (endpoint 2.2); the relationship between the achievement of clinical remission (CR) or CR off-GCs at 6 months and the temporal trend across the six AAV-PRO domains (endpoint 2.3).

#### Data analysis

All analyses were performed using the Stata software. A 2-sided *P*-value of 5% was considered statistically significant, with Bonferroni correction for multiple tests applied for post-hoc comparisons. Median and 25th–75th percentiles (IQR) were computed to summarize continuous variables and counts and percent to summarize categorical variables. Categorical variables were dichotomized at their upper tertile for subgroup analysis. BVASv3 was grouped into three categories for endpoint 2.1. The extended Mantel and Haenszel test for ranks was used to compare baseline AAV-PRO scores. The Mann–Whitney *U* test was used to compare baseline PROs scores by PtGA groups (>50 *vs* ≤50). Two-level linear mixed models, with random effects for centre and patient, were used to model the effect of time on AAV-PRO scores (endpoint 1). These models were adjusted for age and sex. For modelling purposes, AAV-PRO scores were log-transformed. Regression coefficients and their 95% confidence intervals (95%CI) were back-transformed to the original scale; quantifying associations as ratios of AAV-PRO scores at each time point *vs* baseline. For endpoint 2.1, models included a term for the biomarkers measured at each time point. For endpoint 2.2, a term for baseline correlates was included. Interaction with time was tested to elicit any effect modification on the time profile of measurement. The same model was fitted for endpoint 2.3. As no qualitative interaction was identified, only main effect models were considered. Partial correlations (95%CI) or ratios (95%CI) were derived and reported. The extended Mantel and Haenszel test for ranks was used to assess differences in clinical outcomes between baseline and 6 months.

## Results

### Demographic and clinical characteristics at baseline

Seventy consecutive patients with EGPA starting treatment with mepolizumab were included in the study, female 54.3%, median age 56 years (48–65). Demographics, clinical and laboratory findings and concomitant treatments are summarized in Table 1. Seventeen (25.4%) and 15 patients (22.4%) had a BVASv3 >4 and a VDI >3 at study enrolment, respectively. Forty-seven (67.1%) and 23 patients (32.9%) received mepolizumab 100 mg and 300 mg/4 weeks, respectively, with the main indication being relapsing/refractory asthma or CRSwNP (95.7% of patients). Patients receiving mepolizumab 100 mg exhibited significantly higher median BVASv3 compared with those on the 300 mg dose [3 (2–5) *vs* 1 (0–4), *P* = 0.002], along with a significantly lower median VDI [2 (1–3) *vs* 3 (2–5), *P* = 0.001]. No other significant differences were observed between the groups.

**Table 1.** Demographics, clinical and laboratory features and treatment-related information of patients with EGPA included in the study

Characteristics	All patients (*n* = 70)
Age, median (IQR), years	56.0 (48.0–65.0)
Female, *n* (%)	38 (54.3)
Relapsing disease, *n* (%)	63 (90%)
Previous vasculitic relapses, *n* (%)	34 (50%)
Previous major relapses, *n* (%)	21 (30.9%)
Disease duration, median (IQR), months	68 (24–120)
BVAS, median (IQR)	3.0 (2–5)
VDI, median (IQR)	2.0 (1–3)

Educational level	

Primary school, *n* (%)	13 (19.7%)
Secondary school, *n* (%)	35 (53.0%)
University, *n* (%)	18 (27.3%)
Not available	4 (5.7%)

Laboratory features	

ANCA positivity at study inclusion, *n* (%)	14 (20%)
MPO-ANCA positivity, *n* (%)	12 (17.1%)
PR3-ANCA positivity, *n* (%)	2 (2.9%)
CRP, median (IQR), mg/L	3.0 (0.9–7.5)
Eosinophil count, median (IQR), *n*/μL	604 (340–1400)

Therapeutic regimes	

Mepolizumab 100 mg/4 weeks, *n* (%)	47 (67.1%)
Mepolizumab 300 mg/4 weeks, *n* (%)	23 (32.9%)
Concomitant GCs, *n* (%)	65 (92.8%)
Concomitant GCs dose, median (IQR), mg/day	10.0 (5–12.5)
Concomitant immunosuppressant, *n* (%)	29 (41.4%)
Azathioprine, *n* (%)	12 (17.1%)
Methotrexate, *n* (%)	10 (14.3%)
Mycophenolate mofetil, *n* (%)	4 (5.7%)
Rituximab, *n* (%)	2 (2.9%)
Leflunomide, *n* (%)	1 (1.5%)

ANCA: anti-neutrophil cytoplasmic antibody; BVAS: Birmingham Vasculitis Activity Score; CRP: C-reactive protein; GCs: glucocorticoids; IQR: interquartile range; MPO: myeloperoxidase; VDI: Vasculitis Damage Index.

### AAV-PRO score and PtGA at baseline

**Figure 1.**
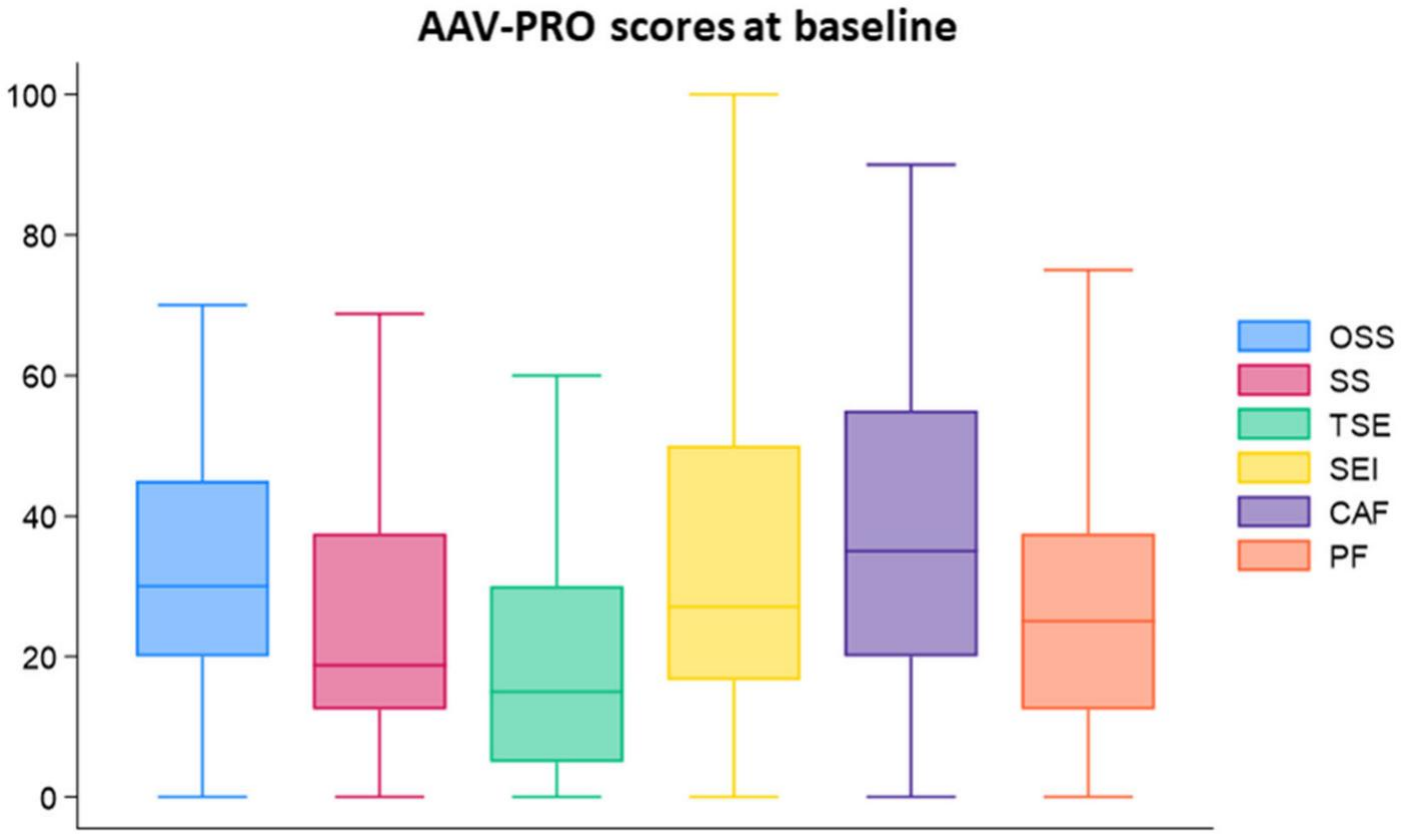
Comparison of the AAV-PRO domains 0–100 score at baseline. AAV-PRO: ANCA-associated vasculitis patient-reported outcomes; CAF: concerns about the future; OSS: organ-specific symptoms; PF: physical function; SEI: social and emotional impact; SS: systemic symptoms; TSE: treatment side effects

The median 0–100 baseline scores of the AAV-PRO domains and PtGA are reported in Fig. 1. A significant heterogeneity between the domains is evident (*P* <0.001), with the highest median scores being 35 for concerns about the future, 30 for organ-specific symptoms, and 27 for social and emotional impact, while the lowest scores were 19 for systemic symptoms and 15 for treatment side effects (Supplementary Table S2, available at *Rheumatology* online). At inclusion, PtGA was 60 (IQR 50–70), with 41 patients (61.2%) experiencing a PtGA >50 on a scale of 0–100. Only the social and emotional impact domain showed statistically significant differences, with median baseline scores of 21 and 37 in patients with PtGA ≤50 and PtGA >50, respectively (Supplementary Table S3, available at *Rheumatology* online).

### Changes over time in the AAV-PROs scores and PtGA during observation period

**Figure 2.**
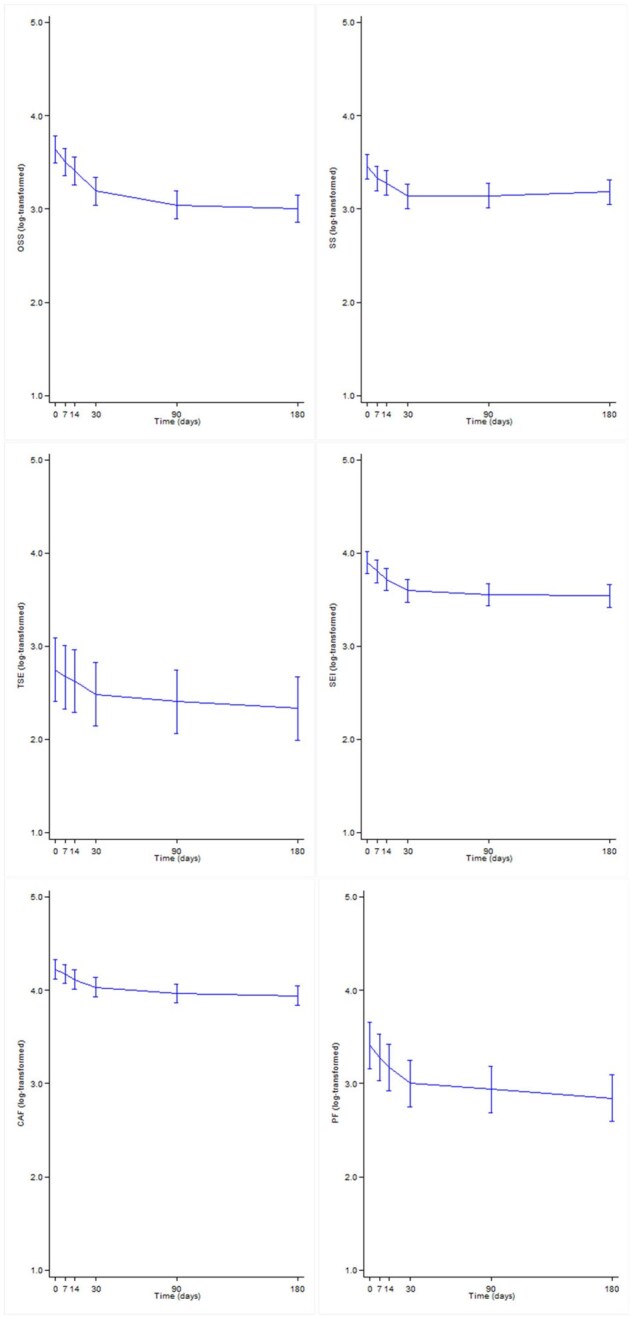
Temporal trend of age- and gender-adjusted log-transformed AAV-PRO domains 0–100 score during the 6-month follow-up. CAF: concerns about the future; OSS: organ-specific symptoms; PF: physical function; SEI: social and emotional impact; SS: systemic symptoms; TSE: treatment side effects

Table 2 and Fig. 2 summarize the changes in AAV-PRO scores and PtGA at each time point. A significant reduction in scores was observed across all domains (*P* <0.001), typically evident by day 14, except for treatment side effects, which showed a significant improvement after 30 days. For PtGA, a significant decrease was observed as early as 7 days later. The ratios of the scores at each time point relative to baseline progressively declined over time, reflecting an improvement in HRQoL. The improvement at 6 months was greatest for the organ-specific symptoms (ratio 0.53), physical function (ratio 0.57) and PtGA (ratio 0.58). At 6 months, the improvement in the other domains was less pronounced, though still significant, with ratios of 0.76, 0.66, 0.70 and 0.76 for systemic symptoms, treatment side effects, social and emotional impact and concerns about the future, respectively.

**Table 2.** Changes over time in log-transformed AAV-PRO domains 0–100 score and PtGA during study observation

TIME	Median (IQR)	Ratio (95%CI)	Time *P*-value	Post-hoc *P*-value
OSS			<0.001	
Baseline	30 (20–45)	Ref.		Ref.
7	20 (15–35)	0.87 (0.74–1.03)		0.253
14	20 (10–30)	0.79 (0.67–0.94)		0.001
30	10 (10–20)	0.64 (0.54–0.75)		<0.001
90	10 (5–20)	0.55 (0.47–0.65)		<0.001
180	5 (0–15)	0.53 (0.45–0.63)		<0.001

		
Post-hoc comparison between consecutive times: 30 vs 14 *P* = 0.002				

SS			<0.001	

Baseline	18.8 (12.5–37.5)	Ref.		Ref.
7	18.7 (6.2–31.2)	0.88 (0.76–1.02)		0.208
14	12.5 (6.2–25)	0.84 (0.72–0.98)		0.01
30	12.5 (6.2–25)	0.73 (0.63–0.85)		<0.001
90	12.5 (0–25)	0.73 (0.63–0.85)		<0.001
180	12.5 (6.3–31.2)	0.76 (0.66–0.89)		<0.001

		
Post-hoc comparison between consecutive times://				

TSE			<0.001	

Baseline	15 (5–30)	Ref.		Ref.
7	10 (5–25)	0.93 (0.75–1.15)		>0.90
14	10 (5–25)	0.89 (0.71–1.10)		>0.90
30	10 (0–20)	0.77 (0.62–0.95)		0.004
90	10 (0–15)	0.71 (0.57–0.88)		<0.001
180	5 (0–15)	0.66 (0.53–0.82)		<0.001

		
Post-hoc comparison between consecutive times://				

SEI			<0.001	

Baseline	27.1 (16.7–50)	Ref.		Ref.
7	25 (12.5–41.7)	0.91 (0.82–1.01)		0.132
14	20.8 (8.3–33.3)	0.83 (0.75–0.92)		<0.001
30	16.7 (4.2–29.2)	0.74 (0.66–0.82)		<0.001
90	12.5 (4.2–29.2)	0.71 (0.63–0.78)		<0.001
180	12.5 (0–33.3)	0.70 (0.63–0.78)		<0.001

		
Post-hoc comparison between consecutive times: 30 vs 14 P = 0.015				

CAF			<0.001	

Baseline	35 (20–55)	Ref.		Ref.
7	30 (15–50)	0.95 (0.88–1.03)		>0.90
14	25 (10–55)	0.90 (0.83–0.97)		0.001
30	25 (10–40)	0.83 (0.77–0.90)		<0.001
90	15 (5–35)	0.77 (0.71–0.84)		<0.001
180	15 (5–30)	0.76 (0.70–0.82)		<0.001

		
Post-hoc comparison between consecutive times://				

PF			<0.001	

Baseline	25 (12.5–37.5)	Ref.		Ref.
7	25 (6.2–37.5)	0.88 (0.73–1.05)		0.472
14	18.7 (6.2–31.2)	0.79 (0.66–0.95)		0.002
30	12.5 (0–25)	0.67 (0.56–0.80)		<0.001
90	12.5 (6.2–18.7)	0.62 (0.52–0.75)		<0.001
180	6.2 (0–25)	0.57 (0.47–0.68)		<0.001

		
Post-hoc comparison between consecutive times://				

PtGA			<0.001	

Baseline	60 (50–70)	Ref.		Ref.
7	50 (30–60)	0.87 (0.79–0.95)		<0.001
14	40 (20–50)	0.83 (0.76–0.91)		<0.001
30	20 (10–40)	0.70 (0.64–0.77)		<0.001
90	20 (10–20)	0.61 (0.56–0.67)		<0.001
180	10 (0–20)	0.58 (0.53–0.63)		<0.001
		
Post-hoc comparison between consecutive times: 30 vs 14 *P*<0.001; 90 vs 30 *P*<0.001				

95%CI: 95% confidence interval; CAF: concerns about the future; IQR: interquartile range; OSS: organ-specific symptoms; PF: physical function; PtGA: patient global assessment; SEI: social and emotional impact; SS: systemic symptoms; TSE: treatment side effects.

Adjusted for age and sex (Bonferroni corrected).

### Association of the AAV-PRO score with PtGA, disease activity and damage accrual over time

The association between the AAV-PRO questionnaire domains scores and PtGA, BVASv3 and VDI, after adjustment for time, is reported in Table 3. PtGA showed a significant correlation with all six AAV-PRO domains scores (*P* <0.001); however, the strength of these associations ranged from low to moderate, with partial correlation coefficients varying from 0.16 for treatment side effects to 0.38 for organ-specific symptoms. BVASv3 was significantly associated with all the AAV-PRO domains scores, except for treatment side effects. In contrast, VDI did not show significant correlations with any of the AAV-PRO questionnaire domain. The ratio of scores for BVASv3 >4 compared with a BVASv3 of 0 was highest for physical function (ratio 2.10) and organ-specific symptoms (ratio 1.65). The relationship between the AAV-PRO domains scores and PtGA, BVASv3 and VDI was independent of time (*P* >0.008 after Bonferroni correction).

**Table 3.** Association of the AAV-PRO score with PtGA, disease activity and damage accrual over time

			Partial correlation	Association depends on time?
	Variable	Partial correlation (95%CI)^b^	*P*-value^a^	*P* for interaction^a^
OSS				

	PtGA	0.38 (0.29–0.46)	<0.001	0.681
	VDI	0.16 (−0.01–0.32)	0.329	0.074
		Ratio (95%CI)^b^	*P*-value^a^	*P* for interaction^a^
	BVAS		<0.001	0.136
	0	Ref	Ref	
	1–4	1.14 (1.01–1.30)	0.046	
	>4	1.65 (1.34–2.04)	<0.001	

SS				

	PtGA	0.17 (0.07–0.26)	<0.001	0.095
	VDI	0.03 (−0.14–0.20)	0.206	0.222
		Ratio (95%CI)^b^	*P*-value^a^	*P* for interaction^a^
	BVAS		0.002	0.326
	0	Ref	Ref	
	1–4	1.13 (1.01–1.28)	0.046	
	>4	1.41 (1.16–1.72)	0.001	

TSE				

	PtGA	0.16 (0.06–0.25)	0.001	0.804
	VDI	0.05 (−0.12–0.22)	0.421	0.328
		Ratio (95%CI)^b^	*P*-value^a^	*P* for interaction^a^
	BVAS		0.091	0.021
	0	Ref	Ref	
	1–4	0.98 (0.82–1.18)	0.870	
	>4	1.32 (0.99–1.77)	0.063	

SEI				

	PtGA	0.30 (0.21–0.39)	<0.001	0.870
	VDI	0.11 (−0.06–0.27)	0.532	0.418
		Ratio (95%CI)^b^	*P*-value^a^	*P* for interaction^a^
	BVAS		0.035	0.397
	0	Ref	Ref	
	1–4	1.06 (0.96–1.16)	0.238	
	>4	1.22 (1.05–1.41)	0.010	

CAF				

	PtGA	0.18 (0.08–0.27)	<0.001	0.725
	VDI	0.07 (−0.10–0.24)	0.627	0.936
		Ratio (95%CI)^b^	*P*-value^a^	*P* for interaction^a^
	BVAS		0.015	0.855
	0	Ref	Ref	
	1–4	1.05 (0.98–1.12)	0,166	
	>4	1.18 (1.05–1.31)	0,004	

PF				

	PtGA	0.30 (0.20–0.39)	<0.001	0.624
	VDI	0.03 (−0.14–0.20)	0.133	0.330
		Ratio (95%CI)^b^	*P*-value^a^	*P* for interaction^a^
	BVAS		<0.001	0.700
	0	Ref	*Ref*	
	1–4	1.29 (1.12–1.48)	<0.001	
	>4	2.10 (1.68–2.63)	<0.001	

a *P*-value Bonferroni correction = 0.008.

b Adjusted for time.

95%CI: 95% confidence interval; BVAS: Birmingham Vasculitis Activity Score; CAF: concerns about the future; IQR: interquartile range; OSS: organ-specific symptoms; PF: physical function; PtGA: patient global assessment; SEI: social and emotional impact; SS: systemic symptoms; TSE: treatment side effects; VDI: Vasculitis Damage Index.

### Subgroup analysis by baseline characteristics, clinical and laboratory features

The association between demographics, clinical and laboratory characteristics and changes in the AAV-PRO domains score over time is reported in Table 4 and further detailed in Supplementary Figs S1–S6, available at *Rheumatology* online. Female patients reported significantly higher scores in terms of systemic symptoms, treatment side effects, social and emotional impact, and concerns about the future (ratios ranging from 0.61 to 0.83). Furthermore, concerns about the future score was significantly higher in patients receiving a GCs dose >10 mg/day at baseline (ratio 1.19), and significantly lower in those treated with concomitant immunosuppressants (ratio 0.83). Higher systemic symptoms values were found in patients with CRP >5 mg/l (ratio 1.33), while treatment side effects score was significantly lower in patients with ≥1 previous vasculitic relapse (ratio 0.62). Conversely, no association with the AAV-PRO domains was found for age, education level, mepolizumab dose, BVASv3, VDI, ANCA and eosinophil count at baseline. No evident qualitative interactions were observed, as the subgroups exhibited similar performance over time.

**Table 4.** Association between baseline demographics, clinical and laboratory characteristics and changes over time in the AAV-PRO score (sub-group analysis)

	Age			Gender			Educational level						Mepolizumab dose			GCs Dose			Concomitant IS		
	≥65 vs <65 years			Males vs females			Secondary vs primary		University vs primary				300 vs 100 mg			>10 vs ≤10 mg			Yes vs No		
Domain	Ratio (95%CI)^a^	*P* ^a^	*P* for interaction^b^	Ratio (95%CI)^a^	*P* ^a^	*P* for interaction^b^	Ratio (95%CI)^a^	*P* ^a^	Ratio (95%CI)^a^	*P* ^a^	Global *P*	*P* for interaction^b^	Ratio (95%CI)^a^	*P* ^a^	*P* for interaction^**^	Ratio (95%CI)^*^	*P* ^*^	***P* for Interaction** ^**^	**Ratio (95%CI)** ^*^	***P*** ^*^	***P* for Interaction** ^**^
OSS	1.14 (0.95–1.36)	0.168	0.874	0.88 (0.74–1.04)	0.137	<0.001	0.92 (0.71–1.17)	>0.90	0.97 (0.73–1.30)	>0.900	0.628	0.447	0.89 (0.71–1.13)	0.357	<0.001	1.02 (0.83–1.25)	0.863	<0.001	0.89 (0.75–1.06)	0.204	0.869
SS	1.22 (0.95–1.58)	0.122	0.276	**0.70 (0.56–0.88)**	**0.002**	0.576	0.83 (0.57–1.20)	0.655	0.92 (0.61–1.40)	>0.900	0.426	0.098	0.92 (0.72–1.18)	0.513	0.652	1.24 (0.96–1.59)	0.099	0.047	0.92 (0.73–1.16)	0.479	0.591
TSE	1.25 (0.86–1.81)	0.246	0.188	**0.61 (0.44–0.85)**	**0.003**	0.088	0.74 (0.44–1.25)	0.515	0.82 (0.45–1.52)	>0.900	0.385	0.320	1.20 (0.73–1.99)	0.472	0.001	1.06 (0.69–1.61)	0.794	0.002	0.78 (0.54–1.12)	0.176	0.341
SEI	1.12 (0.93–1.35)	0.236	0.099	**0.72 (0.62–0.85)**	**<0.001**	0.034	0.84 (0.64–1.10)	0.371	0.85 (0.62–1.16)	0.602	0.288	0.071	0.85 (0.67–1.09)	0.201	0.232	1.16 (0.95–1.42)	0.148	0.008	0.89 (0.74–1.06)	0.201	0.275
CAF	1.06 (0.98–1.24)	0.431	0.083	**0.83 (0.72–0.96)**	**0.010**	0.788	0.86 (0.68–1.07)	0.288	0.83 (0.64–1.07)	0.240	0.172	0.259	1.00 (0.82–1.22)	0.981	0.245	**1.19 (1.01–1.40)**	**0.040**	0.042	**0.83 (0.72–0.96)**	**0.011**	0.012
PF	1.14 (0.89–1.46)	0.290	0.640	0.83 (0.66–1.05)	0.122	0.770	0.72 (0.51–1.02)	0.068	0.72 (0.49–1.08)	0.167	0.064	0.977	0.94 (0.66–1.36)	0.757	0.387	1.18 (0.88–1.56)	0.265	<0.001	0.96 (0.75–1.22)	0.736	0.979

	Previous vasculitic relapses			BVAS			VDI			ANCA			Eosinophil count			C-reactive protein		
	>1 vs ≤1			>4 vs ≤4			>3 vs ≤3			Positive vs negative			>500 vs ≤500/μL			>5 vs ≤5 mg/L		
Domain	Ratio (95%CI)^a^	*P* ^a^	*P* for interaction^b^	Ratio (95%CI)^a^	*P* ^a^	*P* for interaction^b^	Ratio (95%CI)^a^	*P* ^a^	*P* for interaction^b^	Ratio (95%CI)^a^	*P* ^a^	*P* for interaction^b^	Ratio (95%CI)^*^	*P* ^*^	***P* for Interaction** ^**^	**Ratio (95%CI)** ^*^	***P*** ^*^	***P* for Interaction** ^**^
OSS	1.14 (0.92–1.40)	0.229	<0.001	1.02 (0.84–1.25)	0.823	<0.001	1.07 (0.87–1.31)	0.543	0.461	0.88 (0.72–1.09)	0.241	0.152	1.13 (0.95–1.34)	0.183	<0.001	1.13 (0.92–1.38)	0.259	<0.001
SS	1.06 (0.81–1.38)	0.666	0.093	1.06 (0.81–1.39)	0.654	0.155	1.22 (0.93–1.61)	0.158	0.467	0.81 (0.61–1.08)	0.159	0.118	1.04 (0.82–1.32)	0.721	0.760	**1.33 (1.01–1.75)**	**0.042**	0.006
TSE	**0.62** **(0.41–0.96)**	**0.032**	0.083	1.02 (0.66–1.56)	0.937	0.015	1.16 (0.74–1.80)	0.521	0.231	0.87 (0.56–1.35)	0.524	0.812	1.28 (0.89–1.84)	0.176	0.258	1.34 (0.87–2.07)	0.177	0.170
SEI	1.01 (0.81–1.26)	0.922	<0.001	1.07 (0.87–1.32)	0.509	<0.001	1.08 (0.87–1.34)	0.464	0.506	0.90 (0.73–1.12)	0.368	0.136	1.12 (0.93–1.34)	0.231	0.202	1.10 (0.88–1.37)	0.398	0.002
CAF	0.94 (0.79–1.12)	0.506	0.219	1.00 (0.84–1.19)	0.985	0.001	1.06 (0.89–1.27)	0.494	0.823	0.91 (0.76–1.08)	0.279	0.467	1.07 (0.92–1.24)	0.359	0.428	1.07 (0.90–1.29)	0.437	0.029
PF	1.11 (0.83–1.49)	0.491	<0.001	1.02 (0.77–1.36)	0.880	<0.001	1.28 (0.95–1.72)	0.099	0.009	0.83 (0.62–1.12)	0.220	0.144	1.16 (0.91–1.48)	0.224	<0.001	1.13 (0.84–1.52)	0.423	<0.001

Bolded section indicates statistically significant results.

a Adjusted for time.

b Do sub-groups have different trends over time?

95%CI: 95% confidence interval; ANCA: anti-neutrophil cytoplasmic antibodies; BVAS: Birmingham Vasculitis Activity Score; CAF: concerns about the future; GCs: glucocorticoids; IS: immunosuppressant; OSS: organ-specific symptoms; PF: physical function; SEI: social and emotional impact; SS: systemic symptoms; TSE: treatment side effects; VDI: Vasculitis Damage Index.

### Clinical outcomes at 6 months

All patients completed the 6-month follow-up, with optimal treatment compliance. Fifty-five patients (78.6%) were able to achieve CR and 22 (31.4%) discontinued GCs due to persistent CR. During the study, seven patients (10%) reported an exacerbation of asthma and/or CRSwNP, whereas four (5.7%) experienced a vasculitic relapse (two patients with peripheral neuropathy, one with skin purpura, one with scleritis), none of them with poor prognostic features. Supplementary Fig. S7 and Table S4, available at *Rheumatology* online show changes over time in the AAV-PRO based on the achievement of CR and CR off-GCs at 6 months. The achievement of CR at 6 months had minimal impact on the performance of the AAV-PRO scores over time, with no significant differences between patients with or without CR. In contrast, CR off-GCs at 6 months was associated with a better trend of treatment side effects (ratio 0.65) and concerns about the future (ratio 0.80) domains. After 6 months of treatment, we observed a statistically significant reduction in disease activity [median BVASv3 from 3 (IQR2-5) to 0 (IQR 0–0), *P* <0.001] and GC dose [median prednisone-equivalent dose from 10 (IQR 5–12.5) to 3.7 (IQR 0–5) mg/day, *P* <0.001], as well as peripheral eosinophil count [from 604 (IQR 340–1400) to 75 (IQR 50–115)/μL, *P* <0.001] and CRP [from 3 (0.9–7.5) to 0.8 (IQR0.1–2.3) mg/l, *P* <0.001]. Damage accrual remained stable throughout the observation period [median VDI from 2 (IQR 1–3) to 2 (IQR 2–3), *P* = 0.083]. None of the patients required a modification of the dose of mepolizumab during the 6-month follow-up. In addition, no AEs attributed to mepolizumab and requiring treatment discontinuation occurred.

## Discussion

In this multicentre, prospective study we assessed the impact of mepolizumab on HRQoL in EGPA patients using the AAV-PRO questionnaire. By combining clinical outcomes with patient-reported data, our study offers a comprehensive evaluation of mepolizumab’s effectiveness, highlighting its rapid, meaningful effects on patients’ well-being. In our cohort, mepolizumab was associated with a significant and quick improvement in PtGA and most AAV-PRO scores, starting as early as 7 and 14 days of therapy, respectively. These improvements were maintained at 6 months, with statistically significant differences from baseline. The treatment side effects domain required 30 days to show statistically significant amelioration, likely due to its low baseline score and the time needed for GC-sparing benefits to reduce the perceived burden of pharmacological AEs, especially those attributable to chronic GC treatment.

The greatest improvement in HRQoL at 6 months occurred for the organ-specific symptoms and physical function, with smaller but significant effects on remaining domains. These results confirm mepolizumab’s ability to control disease activity, reduce cumulative GCs dose [9–11], and positively affect functional capacity and psychosocial dimension over time. Notably, AAV-PRO scores improved most within the first month of treatment. While these data underscore the drug’s rapidity of action in alleviating symptoms, smaller changes observed between 3 and 6 months likely reflect early achievement of low scores at 1 and 3 months, limiting further improvement potential. Importantly, sensitivity to change and the minimal clinically important difference for the AAV-PRO questionnaire in EGPA are still unclear and warrant future investigation. Evidence supporting the rapidity of biological and clinical effect of mepolizumab is scarce and primarily derived from asthma cohorts [27, 28]. In a recent placebo-controlled trial focused on eosinophil kinetics of 20 patients with moderate-to-severe asthma, mepolizumab 100 mg/4 weeks promoted a significant decline in blood and sputum eosinophils after 2 and 42 days, respectively [29]. The MIRRA phase 3 trial, investigating mepolizumab 300 mg/4 weeks in relapsing and/or refractory EGPA, reported significant improvement in the Asthma Control Questionnaire and Sino-nasal Outcome Test-22 after 1–4 weeks and 12 weeks, respectively, compared with placebo [9]. However, no prior study in EGPA evaluated the impact of specific treatments on PROs using the AAV-PRO questionnaire, which, being disease-specific, provides a more holistic depiction of HRQoL in AAV.

The psychosocial implications of the disease and its treatment significantly influence the patients’ perception of symptom burden [30, 31], as reflected by relatively high baseline concerns about the future and social and emotional impact scores and their less pronounced improvement in our study. Supporting this, a recent German study by Maunz *et al.* demonstrated strong correlations between all AAV-PRO domains and Beck’s depression inventory and the Short Form-36 questionnaire in AAV; conversely, no significant associations were observed with BVASv3 or VDI [32]. In our study, a low-to-moderate yet statistically significant correlation was observed between all AAV-PRO domains and PtGA during follow-up. Our results highlight the AAV-PRO questionnaire’s validity for capturing the patients’ perspectives before and after treatment modifications. Except for the treatment side effects domain, all AAV-PRO domains positively correlated with BVASv3. This finding strengthens the importance of shared decision-making, allowing clinicians to discuss the implications of disease activity not just in clinical terms but also in relation to the patient’s quality of life. Consistent with Maunz’s findings, no significant correlation was found between any AAV-PRO domain and VDI. Individual differences in health perception and adaptation to chronic conditions may explain discrepancies between damage assessment and PROs.

Subgroup analyses investigating the influence of baseline characteristics on the performance of specific AAV-PRO domains revealed no differences among most subgroups. A notable exception was female sex, which was significantly associated with higher systemic symptoms, treatment side effects, social and emotional impact, and concerns about the future scores. Our findings align with the validation study of the AAV-PRO questionnaire [19] and two additional studies evaluating the distribution of the questionnaire in Mexican and Italian AAV population [33, 34]. Similarly to other conditions, poorer PROs have been reported in female patients with AAV [35], potentially reflecting both biologic and psychosocial mechanisms. Gender differences in HRQoL should guide healthcare providers to tailor treatment strategies to the complexity of the patients’ experiences. Concerns about the future domain scores were significantly higher in patients on GCs dose >10 mg/day at baseline and significantly lower in those receiving immunosuppressants. These findings reflect the patients’ increased awareness of AEs associated with chronic GC use, as well as the sense of relief that may arise from the availability of a GC-sparing agent. Interestingly, no significant impact from age, educational level, CRP, eosinophil count, ANCA status or mepolizumab dose on the trend of the AAV-PRO questionnaire during the 6-month follow was observed. For mepolizumab dose, our findings align with emerging real-world evidence supporting the potential effectiveness of low-dose mepolizumab in managing EGPA [12, 36], though further research is needed to validate these observations. Importantly, no patient required dosage adjustments or treatment discontinuation due to AEs, confirming the good safety profile of mepolizumab in EGPA.

Our study has a number of strengths. Firstly, we prospectively investigated the impact and the rapidity of mepolizumab’s effect on PtGA and the AAV-PRO questionnaire, as well as the predictors of response of PROs in a longitudinal cohort of patients with EGPA. Moreover, our relatively large population included patients from four different European countries, adding geographical heterogeneity that enhances the generalizability of our findings. The use of a disease-specific tool, such as the AAV-PRO questionnaire, ensures that patients’ perspectives are thoroughly captured. Importantly, our study focused on EGPA, a subtype of AAV underrepresented in prior research using the AAV-PRO questionnaire. However, limitations include its observational nature and the absence of a control group. Secondly, we cannot entirely exclude that changes in GC dose prescribed more than two weeks before the initiation of mepolizumab may have partly contributed to early reductions in some questionnaire domains (e.g. organ-specific symptoms, systemic symptoms). Nonetheless, significant improvements were observed across all questionnaire domains, including those known to be negatively affected by GCs, without further deterioration after GCs tapering or discontinuation. Additionally, it cannot be ruled out that the initial benefits observed in PROs may have been somewhat overestimated due to the subjective and psychological relief associated with the start of a new therapy. Although all versions of the AAV-PRO used in this study were developed through standardized translation procedures, we acknowledge the potential for minor residual cultural or linguistic bias across our multinational cohort.

In conclusion, in our cohort of patients with EGPA, mepolizumab was associated with significant and quick improvements in HRQoL, with positive implications for clinical, functional and psychosocial components. Our findings reinforce mepolizumab’s role as a valuable therapeutic option for EGPA, especially for patients with relapsing disease or those seeking to minimize long-term GCs use. Integrating the AAV-PRO questionnaire into daily practice may improve care quality and foster patient engagement towards a precision-medicine approach. Future trials and long-term studies are warranted to fully explore the properties of the AAV-PRO questionnaire in different AAV populations.

## Supplementary Material

keaf232_Supplementary_Data

## Data Availability

Data available on request: the data underlying this article will be shared on reasonable request to the corresponding author.
