# Supplementary material for: Impact of mepolizumab on the AAV-PRO questionnaire in eosinophilic granulomatosis with polyangiitis: data from a European multicentre study
Source: Rheumatology (Oxford). 2025 Apr 26;64(9):4937–47. doi: 10.1093/rheumatology/keaf232 (PMC12407232; doi:10.1093/rheumatology/keaf232)

Supplementary Table S1. The AAV PRO is a profile measure of 29 items over six domains

| **Organ- Specific symptoms (OSS)** | **Systemic symptoms (SS)** | **Treatment Side Effects (TSE)** |
| --- | --- | --- |
| - Problems with nose and sinuses - Problems with ears - Chest problems - Problems with mouth or throat - Problems with eyes | - Muscle pains, cramps and weakness - Problems with joints - Feeling uncomfortably hot, cold or feverish - Tiredness or fatigue | - Felt embarrassed or self-conscious about appearance/ symptoms - Felt concern about weight - Getting enough good sleep - Indigestion, heartburn, nausea - Skin problems |
| **Social and Emotional Impact (SEI)** | **Concerns about the Future (CAF)** | **Physical Function (PF)** |
| - Been anxious, worried, stressed - Felt down or depressed - Difficulty concentrating or being focused - Felt that I have let people down - Felt life is now focused on coping with my condition - Felt upset/ frustrated as unable to work or do everyday tasks | - Difficulty making long-term plans   Worried about….   - …being dependent on other people - …what will happen to me in the future - …travelling a long distance from home - …long-term effects of treatment | - Walking around shops for an hour - Walking up a flight of stairs - Doing physical activities that you wanted to do - Washing and drying yourself or getting dressed. |

Supplementary Table S2. Comparison of the AAV-PRO 0—100 score at baseline

| **AAV-PRO domain** | **Median (IQR)** | **Extended Mantel-Haenszel  p-value** | **Post-hoc p-value<0.003 (Bonferroni correction)** |
| --- | --- | --- | --- |
|  |  | <0.001 |  |
| OSS | 30 (20-45) |  | vs TSE |
| SS | 18.7 (12.5-37.5) |  | vs CAF |
| TSE | 15 (5-30) |  | vs SEI, CAF, PF, OSS |
| SEI | 27.1 (16.7-50) |  | vs TSE |
| CAF | 35 (20-55) |  | vs PF, TSE, SS |
| PF | 25 (12.5-37.5) |  | vs CAF, TSE |
| PtGA | 60 (50-70) |  |  |

IQR: interquartile range; OSS: organ-specific symptoms; SS: systemic symptoms; TSE: treatment side effects; SEI: social and emotional impact; CAF: concerns about the future; PF: physical function; PtGA: patient global assessment

Supplementary Table S3. Comparison of the AAV-PRO baseline 0—100 score according to baseline PtGA

| **Domain** | **PtGA ≤50** | **PtGA >50** | **Mann Whitney U test** |
| --- | --- | --- | --- |
|  | **Median (IQR)** | **Median (IQR)** | **p-value** |
| OSS | 30 (20-40) | 25 (20-45) | 0,796 |
| SS | 18.7 (12.5-31.2) | 25 (12.5-37.5) | 0,357 |
| TSE | 10 (0-15) | 15 (5-30) | 0,118 |
| SEI | 20.8 (12.5-29.2) | 37.5 (20.8-54.2) | **0,016** |
| CAF | 35 (20-45) | 40 (20-60) | 0,592 |
| PF | 18.7 (12.5-37.5) | 31.2 (18.7-50) | 0,08 |

IQR: interquartile range; OSS: organ-specific symptoms; SS: systemic symptoms; TSE: treatment side effects; SEI: social and emotional impact; CAF: concerns about the future; PF: physical function; PtGA: patient global assessment

Bolded section indicates statistically significant results

Supplementary Table S4. Association between clinical remission and clinical remission off-GCs at 6 months and changes over time in the AAV-PRO score (sub-group analysis)

|  | **Clinical Remission** | | | **Clinical remission off-GCs** | | |
| --- | --- | --- | --- | --- | --- | --- |
|  | **Yes vs No** | | | **Yes vs No** | | |
| **Domain** | **Ratio (95%CI)*** | **p*** | ***P for Interaction***** | **Ratio (95%CI)*** | **p*** | ***P for Interaction***** |
| **OSS** | 0.91 (0.75-1.11) | 0,338 | *0,994* | 1.06 (0.88-1.29) | 0,534 | *0,002* |
| **SS** | 0.79 (0.60-1.05) | 0,101 | *0,101* | 0.91 (0.71-1.17) | 0,453 | *0,591* |
| **TSE** | 0.73 (0.49-1.09) | 0,123 | *0,645* | **0.65 (0.44-0.96)** | **0,029** | *0,840* |
| **SEI** | 0.90 (0.73-1.10) | 0,297 | *0,212* | 0.85 (0.70-1.04) | 0,110 | *0,286* |
| **CAF** | 0.98 (0.83-1.16) | 0,800 | *0,151* | **0.80 (0.69-0.94)** | **0,005** | *0,510* |
| **PF** | 0.86 (0.65-1.13) | 0,268 | *0,505* | 0.86 (0.66-1.13) | 0,276 | *0,665* |

OSS: organ-specific symptoms; SS: systemic symptoms; TSE: treatment side effects; SEI: social and emotional impact; CAF: concerns about the future; PF: physical function; 95%CI: 95% confidence interval; GCs: glucocorticoids.

* Adjusted for time

** Do sub-groups have different trends over time?

Bolded section indicates statistically significant results

Supplementary Figure S1. Association between baseline clinical findings, clinimetric indexes and laboratory findings and changes in organ-specific symptoms domain score over time


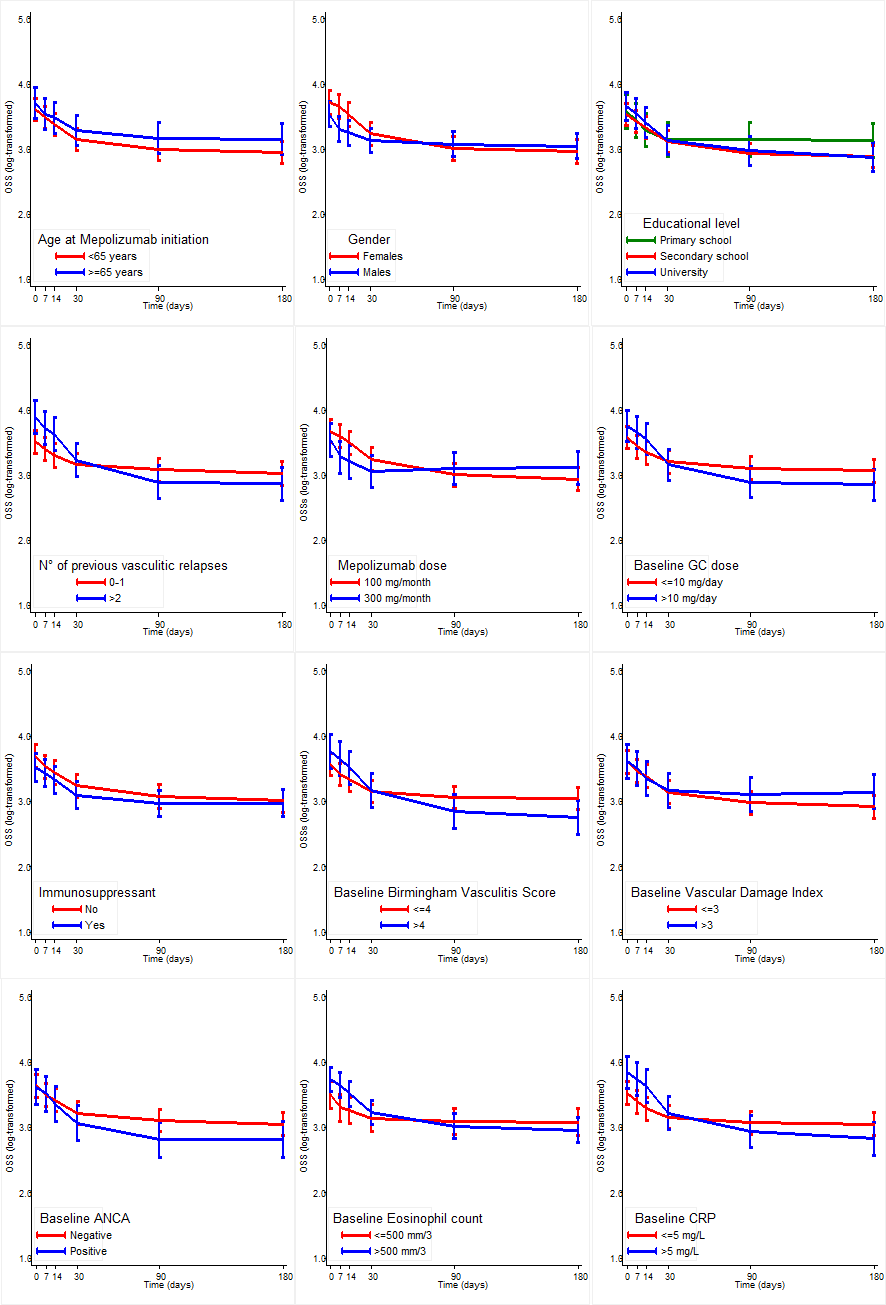


Supplementary Figure S2. Association between baseline clinical findings, clinimetric indexes and laboratory findings and changes in systemic symptoms domain score over time


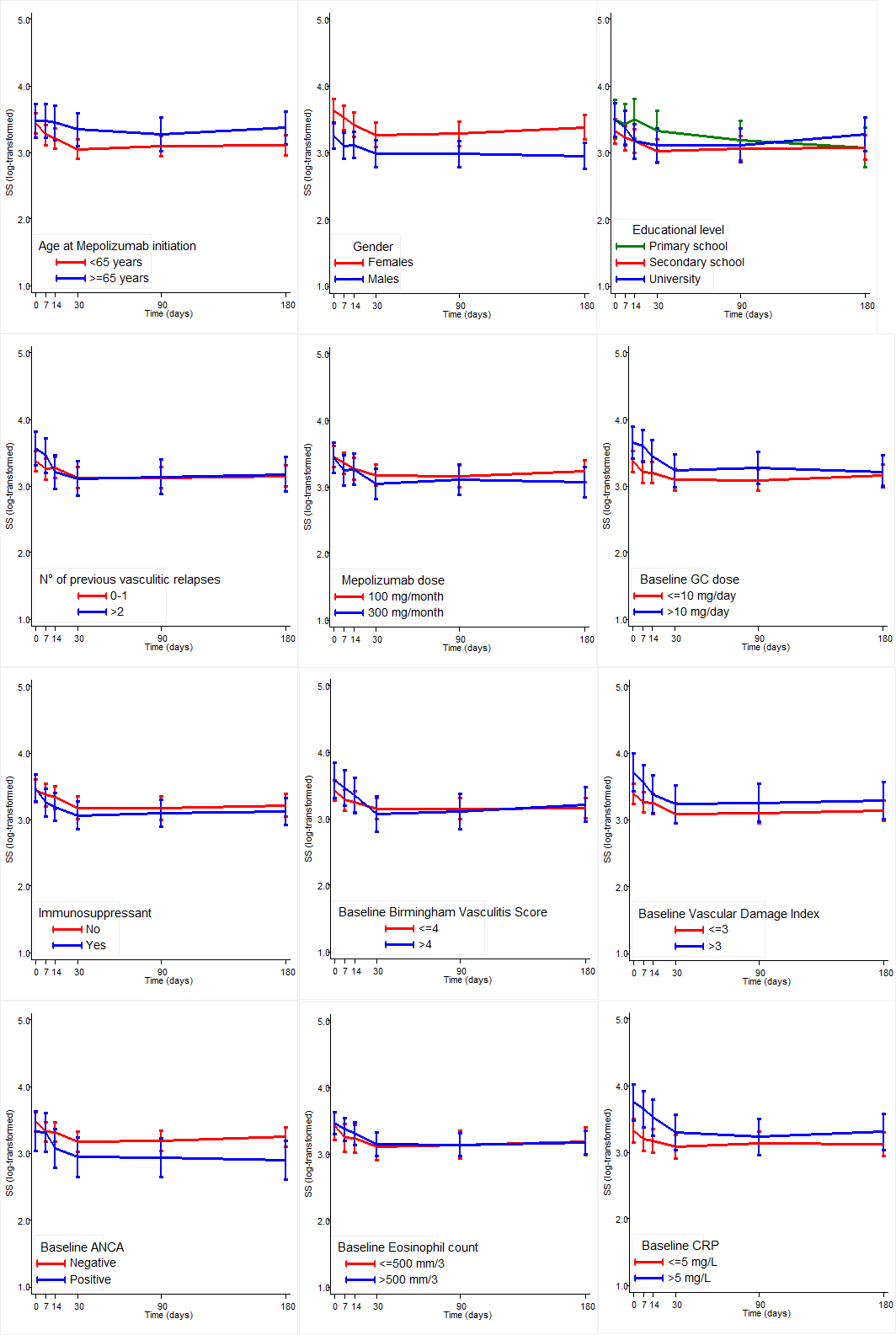


Supplementary Figure S3. Association between baseline clinical findings, clinimetric indexes and laboratory findings and changes in treatment side effects domain score over time.


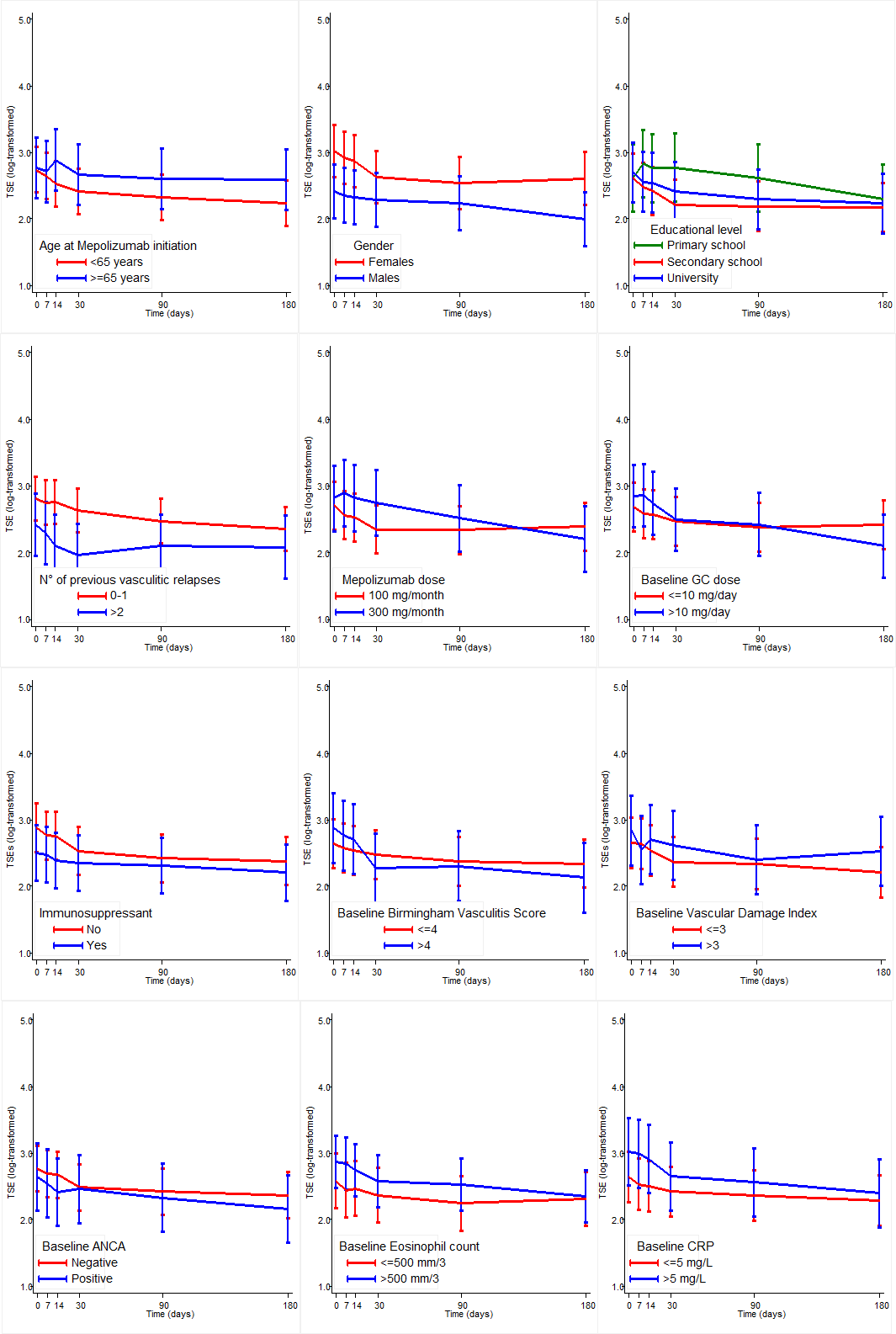


Supplementary Figure S4. Association between baseline clinical findings, clinimetric indexes and laboratory findings and changes in social and emotional impact domain score over time.


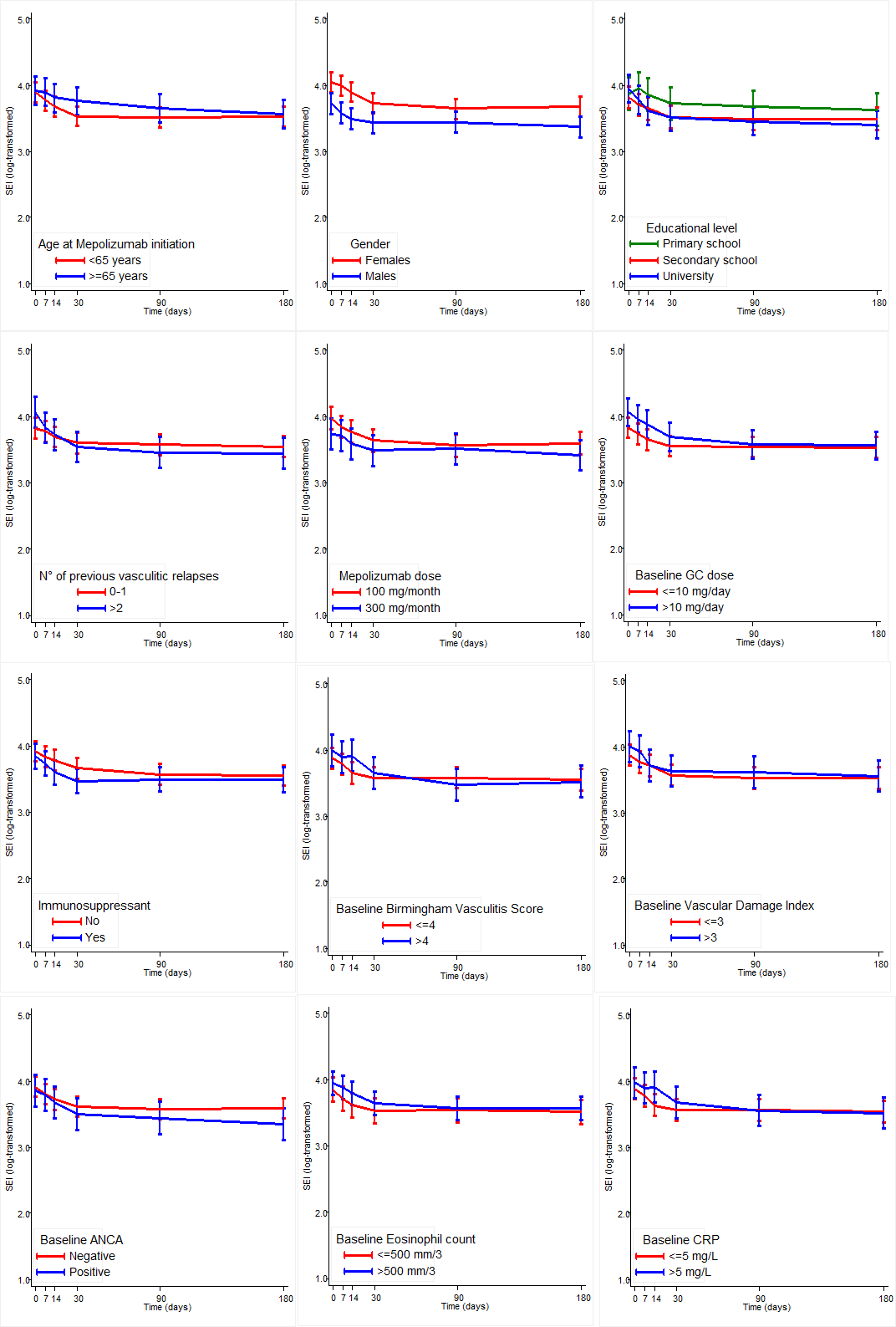


Supplementary Figure S5. Association between baseline clinical findings, clinimetric indexes and laboratory findings and changes in concerns about the future domain score over time.


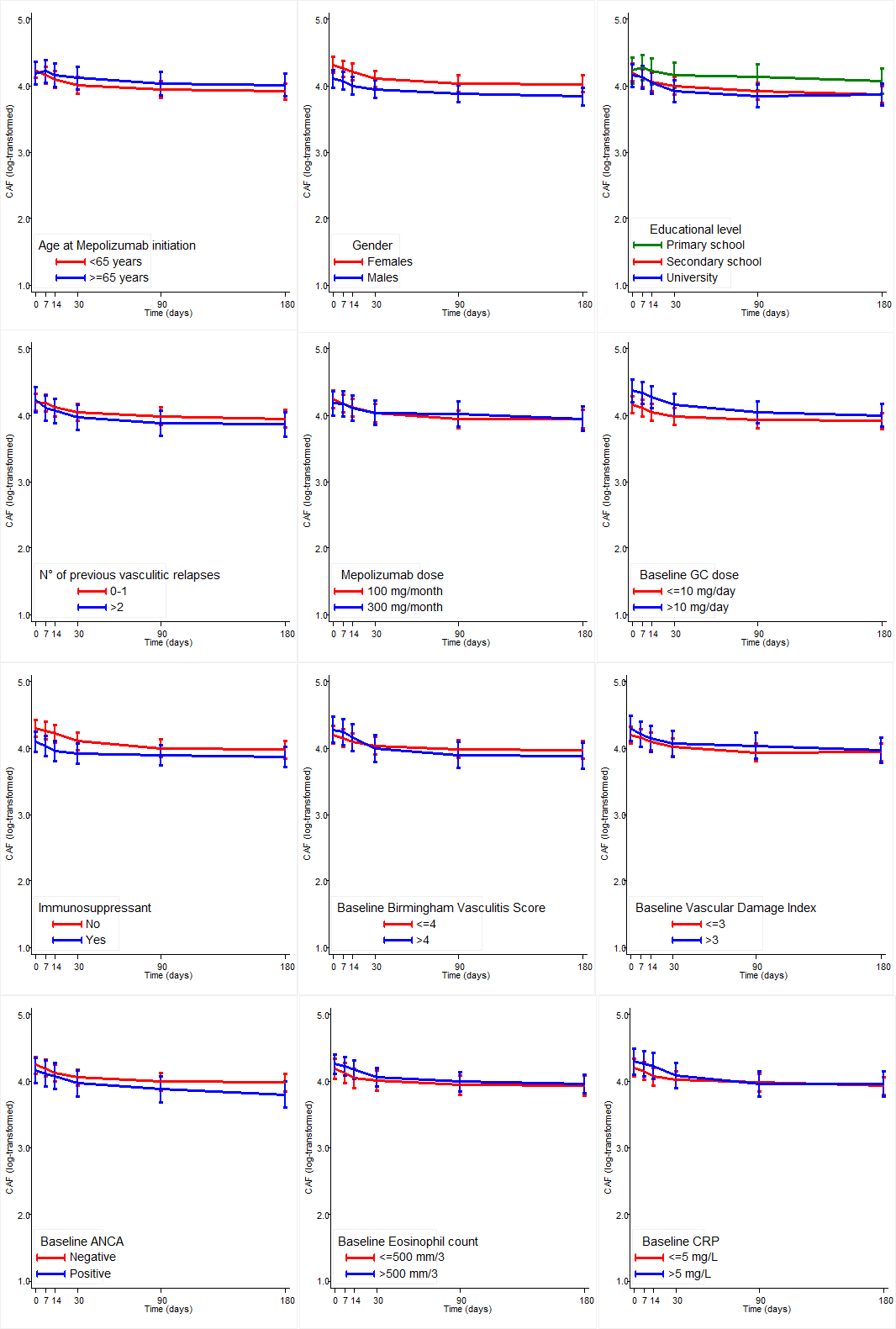


Supplementary Figure S6. Association between baseline clinical findings, clinimetric indexes and laboratory findings and changes in physical function domain score over time.


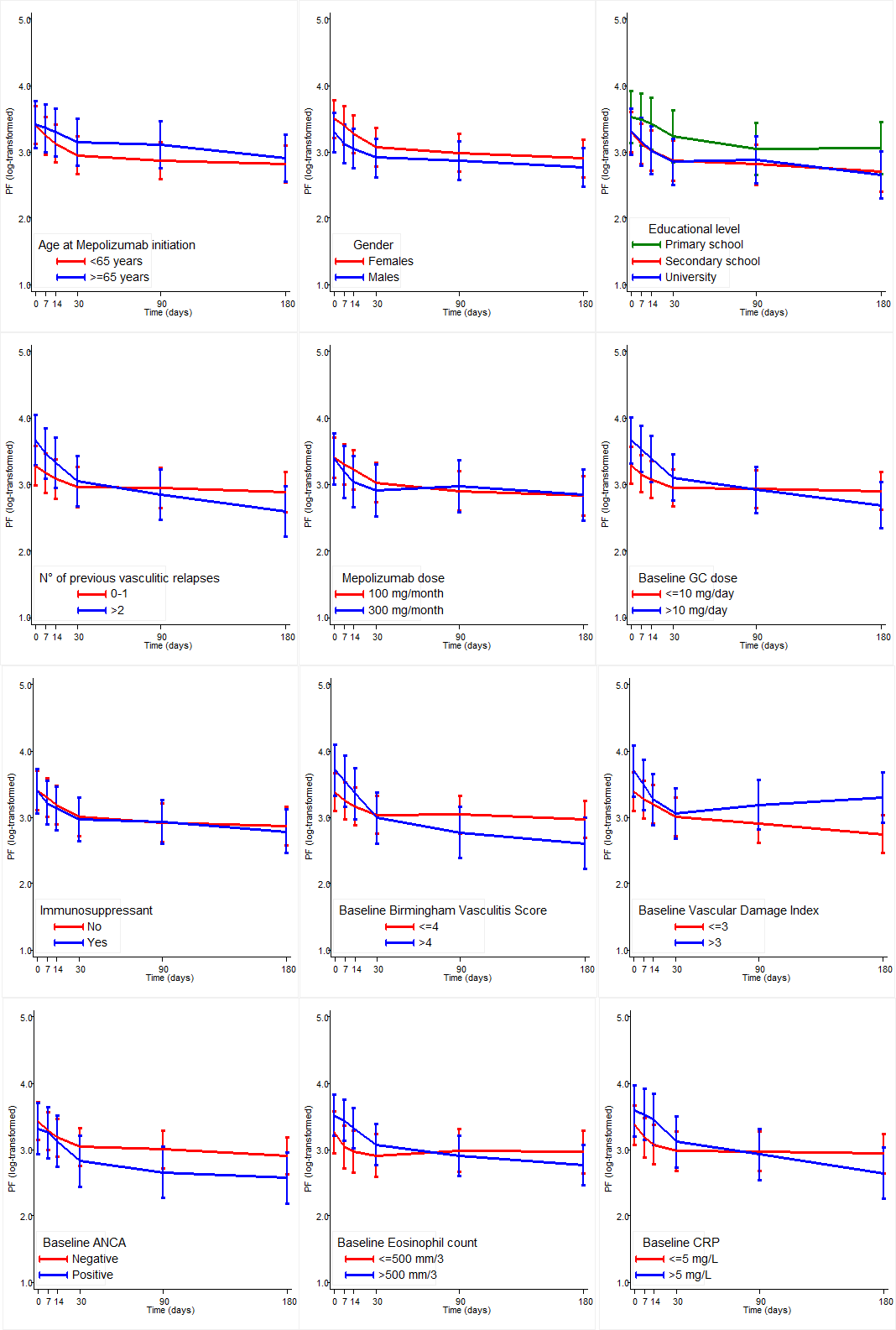


Supplementary Figure S7. Association between changes over time in the AAV-PRO domains and the achievement of clinical remission (Panel A) and clinical remission off-glucocorticoids (Panel B) at 6 months


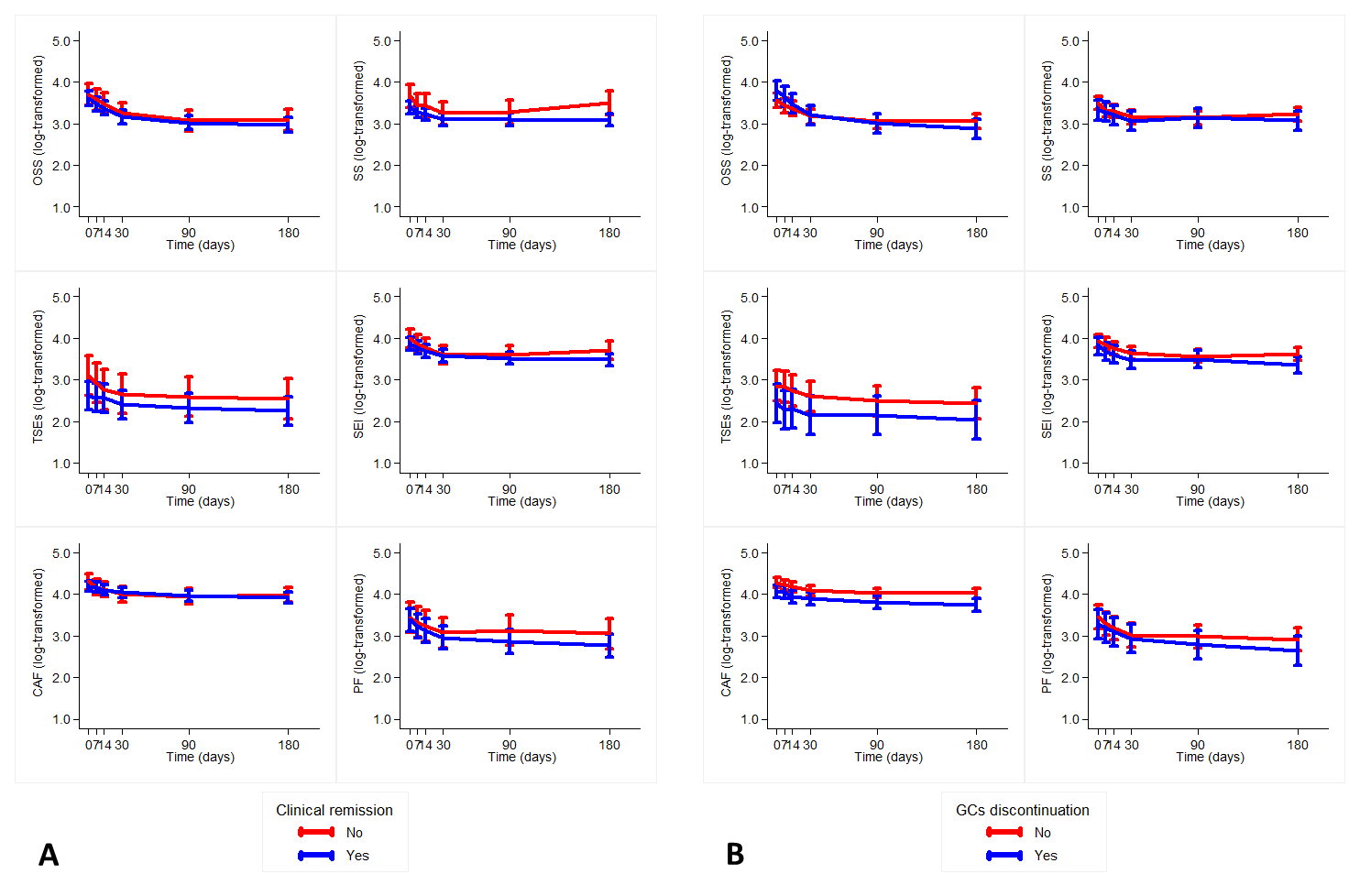

Supplement: keaf232_Supplementary_Data [file keaf232_supplementary_data.docx]
